# Design, biological evaluation and 3D QSAR studies of novel dioxin-containing pyrazoline derivatives with thiourea skeleton as selective HER-2 inhibitors

**DOI:** 10.1038/srep27571

**Published:** 2016-06-08

**Authors:** Bing Yang, Yu-Shun Yang, Na Yang, Guigen Li, Hai-Liang Zhu

**Affiliations:** 2 State Key Laboratory of Pharmaceutical Biotechnology, Nanjing University, Nanjing 210093, China; 1 Institute of Chemistry and BioMedical Sciences, School of Chemistry and Chemical Engineering, Nanjing University, Nanjing 210023, China

## Abstract

A series of novel dioxin-containing pyrazoline derivatives with thiourea skeleton have been designed, synthesized and evaluated for their EGFR/HER-2 inhibitory and anti-proliferation activities. A majority of them displayed selective HER-2 inhibitory activity against EGFR inhibitory activity. Compound **C20** displayed the most potent activity against HER-2 and MDA-MB-453 human breast cancer cell line (IC50 = 0.03 *μ*M and GI50 = 0.15 *μ*M), being slightly more potent than the positive control Erlotinib (IC50 = 0.16 *μ*M and GI50 = 1.56 *μ*M) and comparable with Lapatinib (IC50 = 0.01 *μ*M and GI50 = 0.03 *μ*M). It is a more exciting result that **C20** was over 900 times more potent against HER-2 than against EGFR while this value was 0.19 for Erlotinib and 1.00 for Lapatinib, indicating high selectivity. The results of docking simulation indicate that the dioxin moiety occupied the exit of the active pocket and pushed the carbothioamide deep into the active site. QSAR models have been built with activity data and binding conformations to begin our work in this paper as well as to provide a reliable tool for reasonable design of EGFR/HER-2 inhibitors in future.

Cancer chemotherapy, due to the progress of medical researches, steps into a new era in which molecular-targeted therapeutic methods bring in higher selective and less toxic agents compared to the old drugs^1^,^2^. Among the receptor protein tyrosine kinases (RPTKs) which is a hot spot^3^, epidermal growth factor receptor (EGFR) kinase has been identified as a critical role in cancer. Although EGFR family comprises four members^4^, deregulation of growth-factor signaling due to hyper-activation of EGFR and HER-2 has been primarily seen in several cancer types^5^,^6^,^7^,^8^. However, EGFR inhibitors face three predicaments: 1) Even EGFR is inhibited, the overexpress of HER-2 can still open the downstream pathway^9^; 2) Point mutation of EGFR exons 19 and 20 may cause reduced sensitivity to drugs^10^; 3) A tremendous downstream pathway may cause toxicity^11^,^12^,^13^. Thus, unique HER-2 inhibitors with high selectivity and low toxicity, instead of pan blockers^14^, become critically significant^15^,^16^,^17^.

The previous 3D-QSAR models of pyrazoline moiety provided a result that three aryl ones suited EGFR better while thiourea backbone fitted HER-2 better relatively in pyrazoline derivatives (Fig. 1)^18^. To acquire compounds remaining HER-2 inhibitory activity but weakening that of EGFR, ring A (3-position group of pyrazoline) should disobey the previous model which meant introducing electron-donating groups at *para* and *meta* positions. Thiourea backbone was reserved also because of its related activity^19^,^20^,^21^. Oxygen heterocyclic ring was introduced due to its reported HER-2 inhibitory activity^22^. Five-member or six-member rings were choices because larger ring meant less potency. However, we have to consider the interference of telomerase inhibiting while using this backbone. Unfortunately EGFR/HER-2 should remain regular independently, thus bi-inhibitor is less recommended than selective ones in such a situation^23^. According to the previous report, compared with five-member ring, six-member one showed weaker telomerase inhibitory activity^24^. Thus, the basic structure of compounds in this paper was settled.

According to the studies above, a novel series of 4,5-dihydro-1*H*-pyrazole derivatives containing thiourea skeleton and dioxin structure was designed as potential HER-2 selective inhibitors and predicted to have a positive progress with a sound cancer therapeutic benefit.

## Results

### Preliminary Calculation

**Figure 2:**
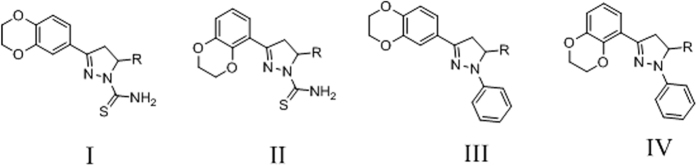
Four series of similar backbones which are potential for exploring selective HER-2 inhibitors.

Before synthesis, the target series was amplified into four series (Fig. 2) to exam their binding energy with EGFR and HER-2, respectively. Molecular docking was conducted to predict the binding energy (CDOCKER INTERACTION ENERGY in CDOCKER protocol in Discovery Studio 3.5) between the four series and EGFR/HER-2. EGFR with Erlotinib (PDB code: 1M17)^25^ and HER-2 with ligand 03Q (PDB code: 3PP0)^26^ were used respectively. The average EGFR binding energy of series I, II, III, IV were −36.3199 kcal/mol, −36.4076 kcal/mol, −40.7584 kcal/mol, −40.4444 kcal/mol. Meanwhile, the top HER-2 binding energy of series I, II, III, IV were −52.8093 kcal/mol, −51.7253 kcal/mol, −50.8926 kcal/mol, −49.7947 kcal/mol. Aiming at acquiring selective HER-2 inhibitors, we prefer Series I after we additionally compared the bottom HER-2 binding energy of series I and II (−40.9818 kcal/mol vs −37.4009 kcal/mol).

**Figure 3:**
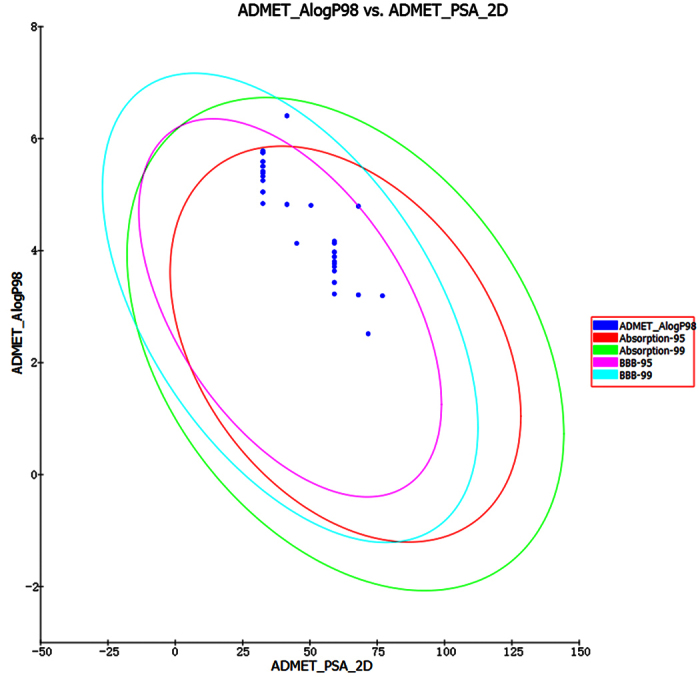
ADMET properties predicted for the four series eighty compounds. Compounds located inside the innermost oval are better for this parameter. The eight compounds outside the innermost oval were No. 6 and No. 16 of the four series with the (benzyloxy)phenyl substitute.

**Figure 4:**
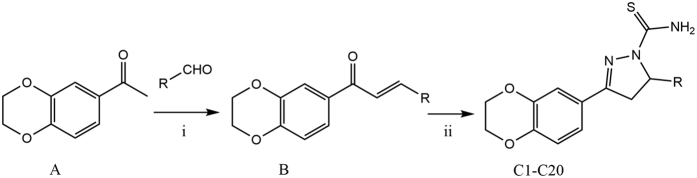
General synthesis of compounds (C1-C20). Reagents and Conditions: i) EtOH, 50% NaOH, reflux, TLC; ii) EtOH, Thiosemicarbazide, 80 °C, overnight.

Since ADMET properties are important parameters of pharmacokinetics^27^,^28^, the ADMET prediction of all 80 compounds was provided (Fig. 3). The results were satisfactory. Thus, we chose Series I (Fig. 4 and Table 1) as target compounds after the preliminary analysis above. The general synthesis method was also organized in Fig. 4.

### Kinase inhibitory activity and cell proliferation

All the synthesized compounds **C1-C20** were evaluated for their EGFR and HER-2 inhibitory activity. The results were expressed as concentrations of IC_50_ (the half maximal inhibitory concentration of EGFR and HER-2 mediated autophosphorylation) and GI_50_ (the half maximal inhibitory concentration of MCF-7 human breast cancer cell line and MDA-MB-453 human breast cancer cell line growth), presented in Table 2. A general glance of the bioactivity as well as SI (the selectivity index of EGFR/HER-2) suggested that most of this series were potential HER-2 inhibitors with merely mediocre EGFR inhibitory activity.

Linear regression (Fig. S1) was conducted to ensure the GI50 values of these compounds shared a similar tendency with their relevant IC50 values, checking that the anti-proliferative effect was produced by inhibitory action against HER-2. As a result, MDA-MB-453 cell line was notably positive correlated to HER-2 (R square = 0.924) while MCF-7 cell line showed no obvious correlation with HER-2 (R square = 0.239). This inference agreed with the relative expression levels of HER-2 in the two cell lines.

Out of the twenty synthesized compounds, **C20** displayed the most potent activity against HER-2 and MDA-MB-453 cell line (IC50 = 0.03 *μ*M and GI50 = 0.15 *μ*M), being comparable with the positive control Erlotinib (IC50 = 0.16 *μ*M and GI50 = 1.56 *μ*M) and Lapatinib (IC50 = 0.01 *μ*M and GI50 = 0.03 *μ*M). A more exciting result was that **C20** was 952 times more potent against HER-2 than against EGFR, because this value was 0.19 for Erlotinib and 1.00 for Lapatinib. This fact indicated that our modification led to favorable effect in both inhibitory activity and selectivity.

### Flow Cytometry

**Figure 5:**
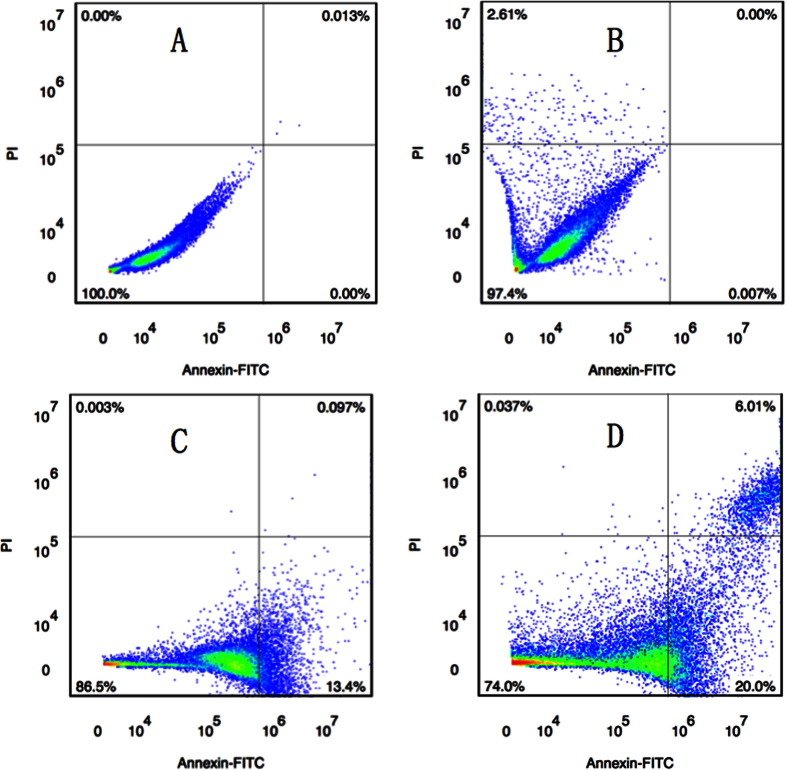
MDA-MB-453 cell line was cultured with various concentrations of **C20** for 24 h. Cells were stained by Annexin V-FITC/PI and apoptosis was analyzed by flow cytometry in a dose-dependent manner. (**A)** Control; (**B**) 0.05 *μ*M; (**C**) 0.1 *μ*M; (**D**) 0.2 *μ*M.

We conducted the flow cytometry (FCM) (Fig. 5) to explain the inhibitory activity of the lead compound **C20**. The results indicated that the compound could induce apoptosis of activated MDA-MB-453 cell line in a dose-dependent manner. With the cells treated with 0.05, 0.1 and 0.2 *μ*M of compounds for 24 h, the percentage of apoptosis by Annexin V-FITC/PI staining was increased correspondingly in a dose-dependent manner.

### Western Blot

**Figure 6:**
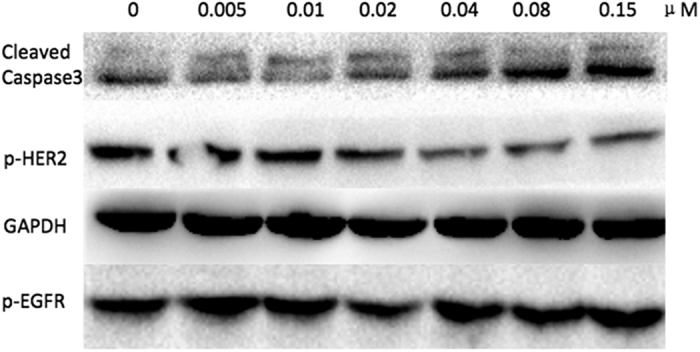
Western blot was performed to detect the effect of compound **C20** on p-HER-2 and p-EGFR.

The corresponding p-HER-2 and p-EGFR levels of MDA-MB-453 dealt by **C20** were measured by Western blot (Fig. 6). With the increasing of **C20** levels, p-HER-2 levels showed gradually trend of decrease. A corresponding increase of Caspase3 levels indicated that this effect could induce apoptosis. Consequently, compound **C20** could directly bind to HER-2, and lower its phosphorylation level. The variation of p-EGFR levels is unconspicuous because the tested concentrations of compound is far below that could cause inhibitory activity of EGFR.

### BLI Assay

**Figure 7:**
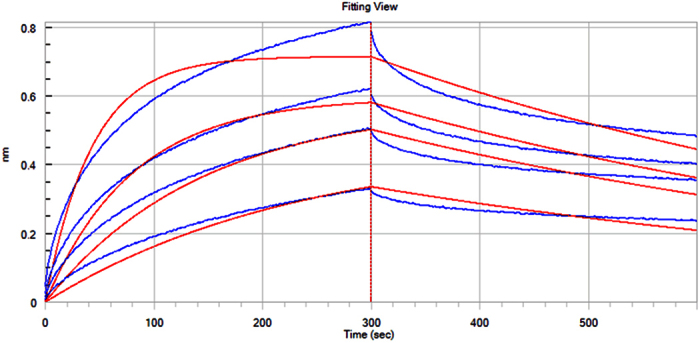
BLI assay of HER-2. Representative time courses of the interference signals elicited by labeled compound **C20**. Data are representative of three separate experiments.

BLI assay was conducted between labeled compound **C20** and HER-2. The result was shown in Fig. 7. The calculated K_D_ by the system itself was 1.87 ± 0.14 × 10^−7^ M, indicating a relatively strong interaction on small molecule level.

### Broadened kinase selectivity

To further expand the discussion of selectivity, we tested the inhibitory activities of representative compounds (**C9**, **C10**, **C13**, **C15**, **C17** and **C20**) against three other related kinases. The three kinases chosen were: VEGFR2 (activating similar pathways and often studied together), HER-3 (also called erbB-3, belonging to EGFR family but activating different pathway), FAK (a downstream kinase of EGFR but in a unique pathway). The results were expressed as IC_50_ values, presented in Table 3. Thus, these results further validated the selectivity of the compounds against HER-2 as well as the positive correlation between bioactivities against HER-2 and MDA-MB-453 (Fig. S2).

### Toxicity test

Besides, the cytotoxic activity of the compounds were evaluated on a mouse embryonic fibroblast cell line (NIH-3T3) using the MTT assay^29^. The results were summarized in Table 4 as well as hemolytic activities. It can be concluded that the selected compounds with potent inhibitory activity and high selectivity were low toxic, which was comparable to the positive control DDCP^30^.

### Docking Study

**Figure 8:**
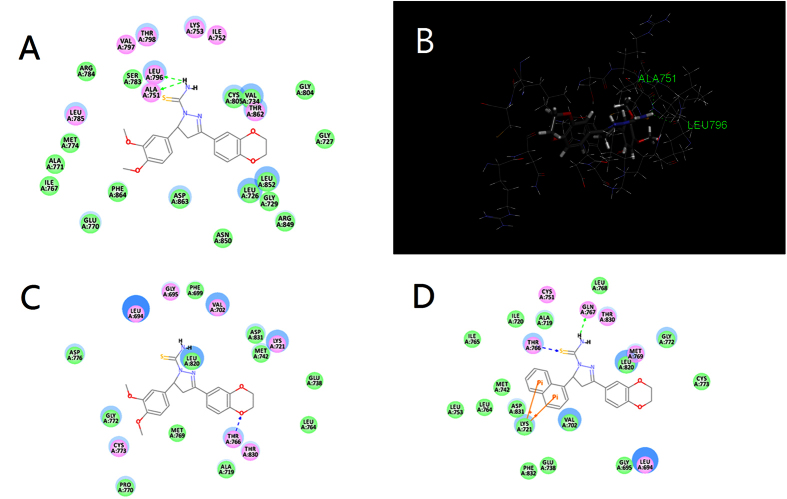
(**A**) 2D molecular docking model of compound **C20** with 3PP0. (**B**) 3D model of the interaction between compound **C20** and 3PP0 binding site. (**C**) 2D molecular docking model of compound **C20** with 1M17. (**D**) 2D molecular docking model of compound **C2** with 1M17. The H-bonds are displayed as dotted lines and the amino acid they act on are labeled in green. The π–cation interactions are shown as orange lines.

Molecular docking is a procedure in which molecular modeling techniques are used to predict how a protein (enzyme) interacts with small molecules (ligands)^31^. Although the CDOCKER protocol in Discovery Studio 3.5 (Discovery Studio 3.5, Accelrys, Inc. San Diego, CA) was used before synthesis to help design our basic backbone, the results could also be used to explore the interaction between compounds and EGFR/HER-2 as well as to visualize the probable binding mode after bioassay. After preparing the receptor and ligands, the site sphere was selected based on the ligand binding location. The binding models of the most potent compounds with corresponding targets were depicted in Fig. 8 (**C20** with 3PP0; **C20** and **C2** with 1M17, respectively).

Not a surprise, the CDocker Interaction Energy (interaction energy between the ligand and the receptor) and the CDocker Energy (energy of the ligand - receptor complexes) agreed with the inhibitory trend for all the synthesized compounds. In the binding model (Fig. 8), compound **C20** is nicely bound to 3PP0 via two hydrogen bonds. One of the amino hydrogen of carbothioamide contributes to both hydrogen bonding interaction with the main chain of ALA751 (N-H^…^O: 2.235 Å, 113.632°) and the main chain of LEU796 (N-H^…^O: 2.242 Å, 132.736°), being a probable explanation for its nice activity. This result indicated that the molecular interactions between both rings of compound and surrounding residues contributed to the strong interaction between the carbothioamide moiety and corresponding residues. When the rings changed, the interactions on the carbothioamide varied. In the EGFR model, compound **C2** is nicely bound to 1M17 via two hydrogen bonds and two π–cation interactions. The sulfur atom of carbothioamide contributes to one of the hydrogen bonding interaction (S^…^H-O: 2.314 Å, 160.269°) with the hydrogen atom on the side chain of THR766. Besides, one of the amino hydrogen of carbothioamide contributes to the hydrogen bonding interaction with the main chain of GLN767 (N-H^…^O: 2.236 Å, 152.083°). Meanwhile, the two π–cation interactions are formed between the naphthalin ring and LYS721 (4.199 Å and 5.490 Å). All these interactions conformed to the relatively nice EGFR inhibitory activity of **C2**. In comparison, however, **C20** is bound to 1M17 via merely one hydrogen bond. The only hydrogen bond is formed between the dioxin ring and side chain of THR766 (O^…^H-O: 2.109 Å, 150.656°). Thus, the binding pattern might suggest the high selectivity of **C20**.

**Figure 9:**
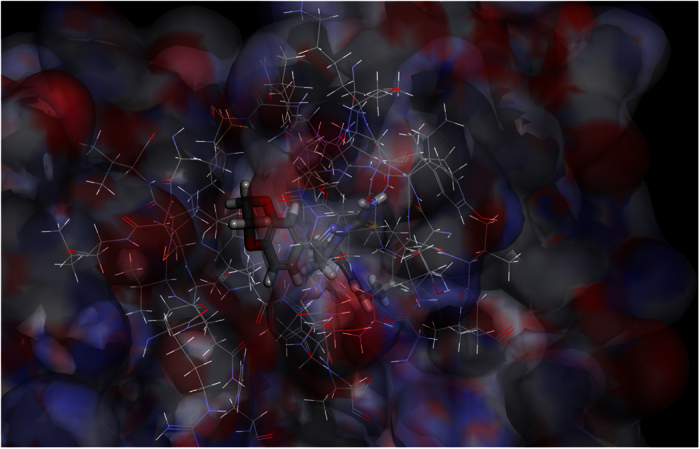
The receptor surface model with **C20** for HER-2.

Moreover, the receptor surface model (Shown in Fig. 9) was conducted to reveal the possible situation of the compounds embedded in the active pocket. The dioxin moiety occupied the exit of the active pocket including VAL734, ALA751, SER783, LEU796, CYS805, LEU852 and THR862, pushed the carbothioamide deep into the active site, confirming the success of structural modification.

### QSAR model

**Figure 10:**
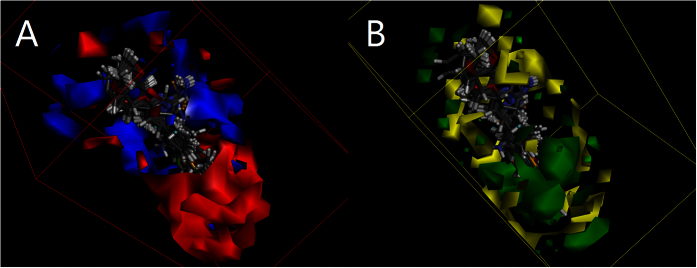
3D-QSAR of 4,5-dihydro-1*H*-pyrazole derivatives containing dioxin moiety with thiourea skeleton for HER-2 (**A,B**). Red contours mean high electron density is expected to increase activity while blue contours mean low electron density is better. Green areas mean steric bulk is better while yellow areas mean small groups are helpful.

By using the Create 3D QSAR protocol of Discovery Studio 3.5, twenty synthesized compounds with definite IC_50_ values against HER-2 were selected as the model dataset. By convention, we used the **pIC**_**50**_ scale (−log IC_50_), in which higher value indicates exponentially greater potency, to measure the inhibitory activity. The training set and testing set were chosen by the Diverse Molecules method in Discovery Studio 3.5. To ensure a good alignment, we chose the alignment conformation of each molecule with lowest energy in the docked results of CDOCKER protocol. Besides, we applied the alignment by the substructure **C1** before building the QSAR model. With the correlation coefficient r^2^ between observed activity of testing set and training set found to be 0.722, the QSAR model we built is acceptable (larger than 0.4). Compounds **C6** and **C16** with -OBn group should be involved otherwise this value could be much higher (Fig. S3). As shown in Fig. 10, the molecules aligned with the *iso*-surfaces of the 3D QSAR model coefficients on electrostatic potential grids and *Van der Waals* grids are listed. Electrostatic map indicates red contours around regions where high electron density (negative charge) is expected to increase activity, and blue contours represent areas where low electron density (partial positive charge) is expected to increase activity. Similarly, steric map indicates areas where steric bulk is predicted to increase (green) or decrease (yellow) activity. As for the dioxin moiety and carbothioamide, they both are in apropriate size, the only point for further modification is that one of the metheylene on dioxin can introduce electron-withdrawing substitute. Meanwhile, the other benzene ring with substituent is more complex. The *para*-position asks for larger group and an electron-donating one is sightly better. Although the meta- and ortho-positions need electron-withdrawing groups, the size should be more strictly controlled within two carbon unit. The 3D QSAR model agrees with the inhibitory activity well and provide us the direction of further modification.

## Discussion

To conclude, a series of novel dioxin-containing pyrazoline derivatives with thiourea skeleton have been designed, synthesized and evaluated for their EGFR/HER-2 inhibitory and anti-proliferation activities. A majority of them displayed selective HER-2 inhibitory activity against EGFR inhibitory activity. Compound **C20** displayed the most potent activity against HER-2 and MDA-MB-453 human breast cancer cell line (IC50 = 0.03 *μ*M and GI50 = 0.15 *μ*M), being slightly more potent than the positive control Erlotinib (IC50 = 0.16 *μ*M and GI50 = 1.56 *μ*M) and comparable with Lapatinib (IC50 = 0.01 *μ*M and GI50 = 0.03 *μ*M). It is a more exciting result that **C20** was over 900 times more potent against HER-2 than against EGFR while this value was 0.19 for Erlotinib and 1.00 for Lapatinib, indicating high selectivity. The results of docking simulation indicated that the dioxin moiety occupied the exit of the active pocket and pushed the carbothioamide deep into the active site. QSAR models were built with activity data and binding conformations to begin our work in this paper as well as to provide a reliable tool for reasonable design of EGFR/HER-2 inhibitors in future.

To deduce how the HER-2 inhibitory activity was affected by the structure variation and modification, subsequently preliminary SAR (Structure Activity Relationship) studies were performed. Initially, we fixed the R group with substituted phenyl group to analyze the substitutes. Benzyloxyphenyl group was temporarily skipped for the extra benzene ring extruded the steric structure backbone but phenyl group was included. For single substitute, *para*-position generally showed better effect than *meta*-position while *ortho*-position nearly ruined the HER-2 inhibitory activity (**C4**, IC50 = 125.3 *μ*M). As for *para*-position, we could perceive the tendency that -OMe > -CF_3_ > -SMe > -Me > -Br > -Cl > -I > -F > -H, inferring that an electron-donating substitute with appropriate size should be involved in designing this kind of inhibitors. The corresponding compounds were **C10** (IC50 = 0.08 *μ*M) > **C17** (IC50 = 0.21 *μ*M) > **C15** (IC50 = 0.29 *μ*M) > **C9** (IC50 = 0.37 *μ*M) > **C13** (IC50 = 0.80 *μ*M) > **C12** (IC50 = 0.84 *μ*M) > **C14** (IC50 = 1.23 *μ*M) > **C11** (IC50 = 1.90 *μ*M) > **C1** (IC50 = 16.01 *μ*M). Thus we could conjecture that appropriate size might be like -OMe or -CF_3_, for the order was -OMe > -CF_3_ > -SMe > -Me instead of -OMe > -SMe > -Me > -CF_3_, and -I was too large, for the order was -Br > -Cl > -I > -F instead of -I > -Br > -Cl > -F. As for *meta*-position, the inferred order was -Br > -Cl > -OMe, indicating that the electronic factor might be more important than the steric factor. The corresponding compounds were **C8** (IC50 = 0.45 *μ*M) > **C7** (IC50 = 62.85 *μ*M) > **C5** (IC50 = 86.55 *μ*M). Meanwhile, for multi substitutes, only two hints could be inferred due to the sample size. One was that 6-F could make up the disadvantage of 2-F to a large extent with the corresponding result **C18** (IC50 = 0.96 *μ*M) > **C4** (IC50 = 125.3 *μ*M). The other was that a *meta*-group with suitable size could indeed contribute to the bioactivity when there already existed a suitable *para*-group, with the corresponding result **C20** (IC50 = 0.03 *μ*M) > **C10** (IC50 = 0.08 *μ*M). Secondly, Naphthalen-1-yl group showed better effect than Naphthalen-2-yl group as **C2** (IC50 = 0.56 *μ*M) > **C3** (IC50 = 7.18 *μ*M). Considering the discussion of substitutes above, the inference was that Naphthalen-2-yl group exceeded the steric limit of *para*- and *meta*- positions while Naphthalen-1-yl group overcome the disadvantage of *ortho-* position by stretching into a larger plane. Thirdly, although a heterocyclic ring (here it was furan) indicated better effect than benzene ring with the corresponding result **C19** (IC50 = 12.04 *μ*M) > **C1** (IC50 = 16.01 *μ*M), the difficulty in introducing substitutes made this feeble superiority fade away. Finally, we came to benzyloxyphenyl group. This group distorted the original backbone no matter the situation was 3-OBn or 4-OBn. Although compounds with benzyloxyphenyl group were not that good as the others in the ADMET properties, both 3-OBn and 4-OBn showed admirable activity accidentally with the corresponding results **C6** (IC50 = 0.06 *μ*M) and **C16** (IC50 = 0.13 *μ*M) respectively. A possible explanation might be the conformational inversion according to the molecular overlap result. That might be an interesting direction in future research.

## Materials and Methods

### Chemistry section

The synthesized compounds **C1-C20** were all prepared in two steps. Firstly, 1-(2,3-dihydrobenzo[*b*][1,4]dioxin-6-yl)ethan-1-one on treatment with different substituted benzaldehydes in presence of 50% NaOH were stirred at room temperature till reactions completed, yielding different analogues of chalcones (**B**). Secondly, thiosemicarbazide was added to participate the cyclization of the obtained analogues of chalcones (**B**), leading to the corresponding target compounds **C1-C20**. All of the synthetic compounds gave satisfactory analytical and spectroscopic data, which were in full accordance with their depicted structures.

(The detailed information is in Supplementary Information).

### Biological Section

#### Ethics statement

This experiments were conducted in accordance with the guideline issued by the State Food and Drug Administration (SFDA of China). The cell lines were cultured and passaged in accordance with the guidelines established by the National Science Council of Republic China. The primary cell lines were purchased from ATCC. All experimental protocols were approved by Academic Committee of Nanjing University.

#### Anti-proliferation Assay

MCF-7 human breast cancer cells was cultured in DMEM/10% fetal bovine serum, in 5% CO_2_ water saturated atmosphere at 37 °C. MDA-MB-453 human breast cancer cells was cultured in L-15 medium in 100% air at 37 °C. Cell suspensions (10000/mL) were prepared and 100 *μ*L/well dispensed into 96-well plates (Costar) giving 1000 cells/well. The plates were returned to the incubator for 24 h to allow the cells to reattach. These compounds were initially prepared at 20 mM in DMSO. Aliquots (200 *μ*L) were diluted into 20 mL culture medium giving 200 *μ*M, and 10 serial dilutions of 3× prepared. Aliquots (100 *μ*L) of each dilution were added to the wells, giving doses ranging from 100 *μ*M to 0.005 *μ*M. After a further incubated at 37 °C for 24 h in a humidified atmosphere with 5% CO_2_, the cell viability was assessed by the conventional 3-(4,5-dimethylthiazol-2-yl)-2,5-diphenyltetrazolium bromide (MTT) reduction assay and carried out strictly according to the manufacturer instructions (Sigma). The absorbance at 590 nm was recorded using LX300 Epson Diagnostic micro-plate reader. Then GI_50_ was calculated using SPSS 13.0 software.

#### EGFR/HER-2 Inhibitory Assay

A 1.7 Kb cDNA encoded for human HER-2 cytoplasmic domain (HER-2-CD, amino acids 676–1245) and 1.6 kb cDNA encoded for the EGFR cytoplasmic domain (EGFR-CD, amino acids 645–1186) were cloned into baculoviral expression vectors pBlueBacHis2B and pFASTBacHTc (Huakang Company China), separately. A sequence that encodes (His)_6_ was located at the 5′ upstream to the HER-2 and EGFR sequences. Sf-9 cells were infected for 3 days for protein expression. Sf-9 cell pellets were solubilized at 0 °C in a buffer at pH 7.4 containing 50 mM HEPES, 10 mM NaCl, 1% Triton, 10 *μ*M ammonium molybdate, 100 *μ*M sodium vanadate, 10 *μ*g/mL aprotinin, 10 *μ*g/mL leupeptin, 10 *μ*g/mL pepstatin, and 16 *μ*g/mL benzamidine, HCl for 20 min followed by 20 min centrifugation. Crude extract supernatant was passed through an equilibrated Ni–NTA superflow packed column and washed with 10 mM and then 100 mM imidazole to remove nonspecifically bound material. Histidine-tagged proteins were eluted with 250 and 500 mM imidazole and dialyzed against 50 mM NaCl, 20 mM HEPES, 10% glycerol and 1 *μ*g/mL each of aprotinin, leupeptin, and pepstatin for 2 h. The entire purification procedure was performed at 4 °C or on ice.

Both EGFR and HER-2 kinase assays were set up to assess the level of autophosphorylation based on DELFIA/Time-Resolved Fluorometry. Compounds **C1-C20** were dissolved in 100% DMSO and diluted to the appropriate concentrations with 25 mM HEPES at pH 7.4. In each well, 10 *μ*L of compound was incubated with 10 *μ*L (12.5 ng for HER-2 or 5 ng for EGFR) of recombinant enzyme (1:80 dilution in 100 mM HEPES) for 10 min at room temperature. Then, 10 *μ*L of 5× buffer (containing 20 mM HEPES, 2 mM MnCl_2_, 100 *μ*M Na_3_VO_4_, and 1 mM DTT) and 20 *μ*L of 0.1 mM ATP-50 mM MgCl_2_ was added for 1 h. Positive and negative controls were included in each plate by incubation of enzyme with or without ATP-MgCl_2_. At the end of incubation, liquid was aspirated and plates were washed three times with wash buffer. A 75 *μ*L (400 ng) sample of europium-labeled anti-phosphotyrosine antibody was added to each well for another 1 h of incubation. After washing, enhancement solution was added and the signal was detected by Victor (Wallac Inc.) with excitation at 340 nm and emission at 615 nm. The percentage of autophosphorylation inhibition by the compounds was calculated using the following equation: 100% - [(negative control)/(positive control - negative control)]. The IC_50_ was obtained from curves of percentage inhibition with eight concentrations of compound. As the contaminants in the enzyme preparation are fairly low, the majority of the signal detected by the anti-phosphotyrosine antibody is from EGFR or HER-2. The experiment was performed for three independent times and in triplicate each time.

#### Flow Cytometry

MDA-MB-453 cell line was seeded per well in 24-well plates and incubated overnight. Then they were treated with compound **C20** at three different concentrations (0.05 *μ*M, 0.1 *μ*M and 0.2 *μ*M, separately). DMSO was chosen as the negative control with a regular dosage of 0.2%. After 24 h, cells were harvested for the apoptosis detection. In brief, collected cells were washed once with PBS and subsequently washed once with binding buffer, and then stained with Annexin V-FITC and propidium iodide (PI) in the binding buffer for 20 min at room temperature in the dark. Apoptotic cells were quantified using a FACScan cytofluorometer (PT. Madagasi Brosa Inc. JI. Batang Hari NO. 73, Propinsi Sumatera Utara, Indonesia) plotting at least 10,000 events per sample. To quantify the data, the frequencies in all quadrants were analyzed using flowjo software. We regarded cells in the lower right quadrant (Annexin V positive/PI negative) as early apoptotic cells, and cells in upper right quadrant (Annexin V positive/PI positive) as late apoptotic cells and necrotic cells.

#### Western Blot

After incubation with compound, cells were harvested and washed with PBS, then lysed in lysis buffer (30 mm Tris, pH 7.5, 150 mm NaCl, 1 mm phenylmethylsulfonyl fluoride, 1 mm Na_3_VO_4_, 1% Nonidet P-40, 10% glycerol, and phosphatase and protease inhibitors). After centrifugation at 12,000 g for 5 min, the supernatant was collected as total protein. The concentration of the protein was determined by a BCATM protein assay kit (Pierce, Rockford, IL, USA). The protein samples were separated by 10% SDS–PAGE and subsequently electrotransferred onto a polyvinylidene difluoride membrane (Millipore, Bedford, MA, USA). The membrane was blocked with 5% non-fat milk for 2 h at room temperature. The blocked membrane was probed with the indicated primary antibodies overnight at 4 °C, and then incubated with a horse radish peroxidase (HRP)-coupled secondary antibody. P-HER-2 and p-EGFR levels were measured as shown in Fig. 6.

#### BLI assay

Bio-layer interferometry (BLI). Binding of compounds (labeled; 0.01, 0.05, 0.1, 0.5 *μ*M) to the HER-2 protein (truncated) was measured and analyzed on an Octet Red96 instrument (ForteBio) at room temperature. The buffer-equilibrated streptavidin biosensors were loaded with 100 *μ*g/mL protein. A duplicate set of sensors was incubated in the buffer without protein for a background binding control. The assay was performed in black 96-well plates (Thermo Fisher Scientific) with the total working volume of 0.21 mL per well. The signal was analyzed using a double reference subtraction protocol to subtract the non-specific binding, background, and signal drift caused by sensor variability. The binding event was quantified by the shift of interference pattern of the light. The result was shown in Fig. 7 and the K_D_ was calculated by the Octet Red96 instrument.

#### HER-3 Inhibitory Assay

A 1.8 Kb cDNA encoded for human HER-3 cytoplasmic domain (HER-3-CD, amino acids 665–1339) was applied to perform the experiment using the same method as EGFR/HER-2.

#### VEGFR-2 Inhibitory Assay

In a 96-well plate, 5 nM/L of either the phosphorylated or nonphosphorylated form of the VEGFR-2 kinase domain was incubated with selected compounds (10-point titration ranging from 4 *μ*M to 0.1 *μ*M), 1 *μ*M/L gastrin substrate, 20 mM/L Tris-HCl, pH 7.5, 10 mM/L MgCl2, 100 mM/L NaCl, 1.5 mM/L EGTA, 1 mM/L DTT, 0.2 mM/L sodium orthovanadate, and 20 *μ*g/mL BSA for 30 min at 25 °C. ATP was added at a final concentration of 11.8 *μ*M and incubated for 60 min at room temperature. Eu-labeled antiphosphotyrosine pT66 antibody (Perkin- Elmer) was then used for detection. The fluorescence at 665 nM was measured with a Wallac Victor plate reader using a time delay of 50 *μ*s. The experiment was performed for three independent times and in triplicate each time.

#### FAK Inhibitory Assay

Selected compounds were tested against FAK. In a typical study, human-recombinant full-length FAK was incubated in kinase buffer containing ATP and the substrate for 4 h at room temperature with or without the presence of the corresponding coumpounds. The final concentration of drug as 100, 30, 10, 3 *μ*g/mL. The remaining ATP in solution was then quantified utilizing the Kinase-Glo-luminescence kit (Promega).

### Safety test section

#### Cytotoxicity test

The cytotoxic activity *in vitro* was measured against mouse fibroblast NIH-3T3 cell using the MTT assay. Cells were cultured in a 96-well plate at a density of 5 × 10^5^ cells and different concentrations of compounds were respectively added to each well. The incubation was permitted at 37 °C, 5% CO_2_ atmosphere for 24 h before the cytotoxicity assessments. 20 *μ*L MTT reagent (4 mg/mL) was added per well 4 h before the end of the incubation. Four hours later, the plate was centrifuged at 1200 rcf for 5 min and the supernatants were removed, each well was added with 200 *μ*L DMSO. The absorbance was measured at a wavelength of 490 nm (OD 490 nm) on an ELISA microplate reader. Three replicate wells were used for each concentration and each assay was measured three times, after which the average of IC_50_ was calculated. The cytotoxicity of each compound was expressed as the concentration of compound that reduced cell viability to 50% (IC_50_). The results were summarized in Table 4.

#### Hemolysis test

Hemolytic activity was assayed using fresh capillary human blood. Erythrocytes were collected by centrifuging the blood three times in chilled phosphate buffered saline (PBS at 4 °C) at 1000 × g for 10 min. The final pellet was resuspended in PBS to give a 2% w/v solution. Using a microtitre plate, 100 *μ*L of the erythrocyte solution was added to dextran, PLL, stearyl-PLL or stearyl-PLL+ LDL (1–1000/*μ*g/mL) in a volume of 100 mL. Samples were then incubated for 3 h and the microtitre plate was centrifuged then at 1000 × g for 10 min and the supernatants (100 *μ*L) transferred into a new microtitre plate. Hemoglobin release was determined spectrophotometrically using a microtitre plate reader (absorbance at 550 nm). Results were expressed as the amount of released hemoglobin induced by the compounds as a percentage of the total. Hemolysis test was tested according to the guide of biological evaluation of medical device (SFDA, China).

### Molecular Modeling

#### Molecular Docking Study

The three-dimensional structures of the aforementioned compounds were constructed using Chem. 3D ultra 12.0 software [Chemical Structure Drawing Standard; Cambridge Soft corporation, USA (2010)], then they were energetically minimized by using MMFF94 with 5000 iterations and minimum RMS gradient of 0.10. The crystal structures of EGFR domain bound to Erlotinib (PDB Code: 1M17.pdb)^25^ and HER-2 domain bound to 03Q (PDB Code: 3PP0.pdb)^26^ complexes were retrieved from the RCSB Protein Data Bank (http://www.rcsb.org/pdb/home/home.do). All bound waters and ligands were eliminated from the protein and the polar hydrogen was added to the proteins. Molecular docking of all twenty-eight compounds was then carried out using the Discovery Stutio (version 3.5) as implemented through the graphical user interface CDocker protocol.

CDOCKER is an implementation of a CHARMm based molecular docking tool using a rigid receptor, including the following steps:

(1) A series of ligands conformations are generated using high temperature molecular dynamics with different random seeds.

(2) Random orientations of the conformations are generated by translating the center of the ligand to a specified position within the receptor active site, and making a series of random rotations. A softened energy is calculated and the orientation is kept when it is less than a specified limit. This process repeats until either the desired number of low-energy orientations is obtained, or the test times of bad orientations reached the maximum number.

(3) Each orientation is subjected to simulated annealing molecular dynamics. The temperature is heated up to a high temperature then cooled to the target temperature. A final energy minimization of the ligand in the rigid receptor using non-softened potential is performed.

(4) For each of the final pose, the CHARMm energy (interaction energy plus ligand strain) and the interaction energy alone are figured out. The poses are sorted according to CHARMm energy and the top scoring (most negative, thus favorable to binding) poses are retained. The whole kinase domain defined as a receptor and the site sphere was selected based on the original ligand binding location, then the original ligand was removed and the ligands prepared by us were placed during the molecular docking procedure. CHARMm was selected as the force field. The molecular docking was performed with a simulated annealing method. The heating steps were 2000 with 700 of heating target temperature. The cooling steps were 5000 with 300 cooling target temperature. Ten molecular docking poses saved for each ligand were ranked according to their dock score function. The pose with the highest -CDocker energy was chosen as the most suitable pose.

#### ADMET Prediction

Absorption, distribution, metabolism, excretion, and toxicity properties (ADMET) of the 20 novel compounds were calculated using the DS software. The aqueous solubility, blood brain barrier penetration, cytochrome P450 2D6 inhibition, hepatotoxicity, human intestinal absorption and plasma protein binding were predicted using this software.

#### QSAR Model

In the model, 80% (that is 16) of the 20 compounds were utilized as a training set for QSAR modeling. The remaining 20% (that is 4) were chosen as test subset using the same protocol. The selected test compounds were: **C5**, **C8**, **C10** and **C15**.

The inhibitory activity of the compounds in literatures [IC_50_ (mol/L)] was initially changed into the minus logarithmic scale [pIC_50_ (mol/L)] and then used for subsequent QSAR analysis as the response variable.

In Discovery Studio, the CHARMm force field is applied and the electrostatic potential together with the *Van der Waals* potential are treated as separate terms. As the electrostatic potential probe, A + le point change is used while distance-dependent dielectric constant is used to mimic the solvent effect. As for the *Van der Waals* potential, a carbon atom with a radius of 1.73 Å is used as a probe.

A Partial Least-Squares (PLS) model is built using energy grids as descriptors. QSAR models were built by using the Create 3D QSAR Model protocol in Discovery Studio 3.5.

### Statistical analysis

Statistical analysis was performed with SPSS Version 13.0 statistic software package. Data were expressed as means ± standard deviation (SD). Comparisons between groups were performed with analysis of non-parametric test. A value of P < 0.05 was considered statistically significant.

## Additional Information

**How to cite this article**: Yang, B. *et al.* Design, biological evaluation and 3D QSAR studies of novel dioxin-containing pyrazoline derivatives with thiourea skeleton as selective HER-2 inhibitors. *Sci. Rep.* **6**, 27571; doi: 10.1038/srep27571 (2016).

## Supplementary Material

Supplementary Information

## Figures and Tables

**Figure 1:**
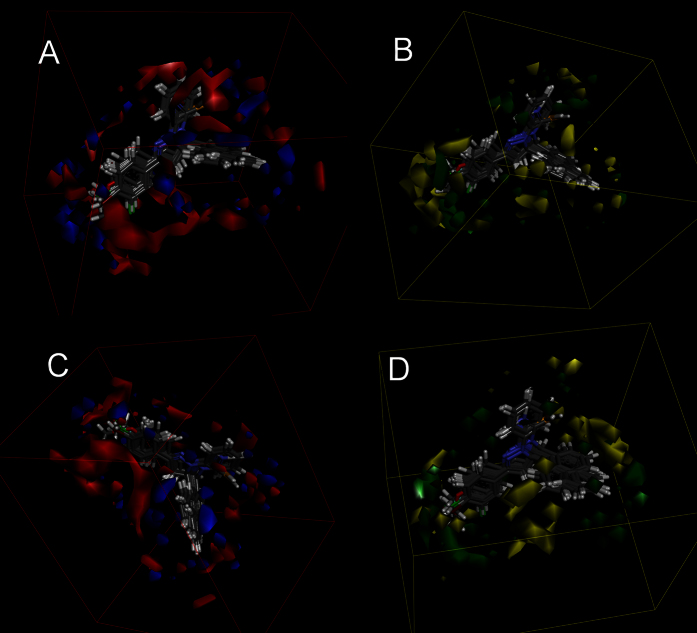
3D-QSAR of 4,5-dihydro-1*H*-pyrazole derivatives containing naphthalin moiety for EGFR (**A,B**) and for HER-2 (**C,D**). Red contours mean high electron density is expected to increase activity while blue contours mean low electron density is better. Green areas mean steric bulk is better while yellow areas mean small groups are helpful.

**Table 1:** Substitutes of the synthesized compounds.

Compound	R	Compound	R
C1	Phenyl	C11	4-Fluorophenyl
C2	Naphthalen-1-yl	C12	4-Chlorophenyl
C3	Naphthalen-2-yl	C13	4-Bromophenyl
C4	2-Fluorophenyl	C14	4-Iodophenyl
C5	3-Methoxyphenyl	C15	4-(Methylthio)phenyl
C6	3-Benzyloxyphenyl	C16	4-Benzyloxyphenyl
C7	3-Chlorophenyl	C17	4-(Trifluoromethyl)phenyl
C8	3-Bromophenyl	C18	2,6-Difluorophenyl
C9	*p*-Tolyl	C19	2-Furanyl
C10	4-Methoxyphenyl	C20	3,4-Dimethoxyphenyl

**Table 2:** EGFR and HER-2 inhibitory activity and selectivity index with anti-proliferation activity of the synthesized compounds (C1-C20).

Compounds	IC50(*μ*M)	IC50(*μ*M)	GI50(*μ*M)	GI50(*μ*M)	SI
	EGFR	HER-2	MDA-MB-453	MCF-7	EGFR/HER-2
C1	21.85 ± 2.03	16.01 ± 1.45	63.4 ± 6.12	10.03 ± 0.91	1.36
C2	2.94 ± 0.26	0.56 ± 0.05	2.20 ± 0.18	4.68 ± 0.42	5.25
C3	4.42 ± 0.35	7.18 ± 0.65	19.9 ± 1.84	5.30 ± 0.49	0.62
C4	59.33 ± 5.58	125.3 ± 10.2	>300	185.4 ± 17.8	0.47
C5	43.58 ± 4.10	86.55 ± 8.05	135.1 ± 11.1	57.2 ± 5.36	0.50
C6	4.49 ± 0.41	0.06 ± 0.004	0.20 ± 0.02	4.95 ± 0.43	74.8
C7	9.45 ± 0.88	62.85 ± 5.67	>300	16.8 ± 0.98	0.15
C8	8.63 ± 0.78	0.45 ± 0.04	1.69 ± 0.15	5.64 ± 0.52	19.2
C9	65.16 ± 5.88	0.37 ± 0.03	1.38 ± 0.12	56.2 ± 5.41	176.1
C10	37.95 ± 3.75	0.08 ± 0.006	0.25 ± 0.02	18.2 ± 1.71	474.4
C11	47.83 ± 4.54	1.90 ± 0.15	11.9 ± 1.05	28.5 ± 2.80	25.2
C12	53.82 ± 5.12	0.84 ± 0.07	5.31 ± 0.48	33.9 ± 3.21	64.1
C13	82.03 ± 7.84	0.80 ± 0.07	3.24 ± 0.30	123.3 ± 11.2	102.5
C14	84.26 ± 7.81	1.23 ± 0.10	9.12 ± 0.89	134.7 ± 11.6	68.5
C15	27.50 ± 2.46	0.29 ± 0.02	0.95 ± 0.08	12.1 ± 1.17	94.8
C16	10.20 ± 0.94	0.13 ± 0.01	0.36 ± 0.03	5.99 ± 0.55	78.5
C17	79.71 ± 7.01	0.21 ± 0.02	0.55 ± 0.02	96.4 ± 9.22	379.6
C18	34.03 ± 3.08	0.96 ± 0.08	8.68 ± 0.84	15.2 ± 1.40	35.4
C19	196.5 ± 18.5	12.04 ± 1.02	33.4 ± 2.95	>300	16.3
C20	28.56 ± 2.52	0.03 ± 0.002	0.15 ± 0.01	11.9 ± 1.06	952.0
Erlotinib	0.03 ± 0.003	0.16 ± 0.01	1.56 ± 0.14	0.08 ± 0.006	0.19
Lapatinib	0.01 ± 0.001	0.01 ± 0.001	0.03 ± 0.002	3.12 ± 0.24	1.00

**Table 3:** Kinases inhibitory activities (VEGFR2, FAK and HER-3) of representative compounds (C9, C10, C13, C15, C17 and C20).

Compounds	IC50(*μ*M)	IC50(*μ*M)	IC50(*μ*M)
	VEGFR-2	FAK	HER-3
C9	11.5 ± 1.07	36.1 ± 3.21	6.32 ± 0.58
C10	6.91 ± 0.64	64.0 ± 5.95	10.7 ± 0.96
C13	25.8 ± 2.41	36.5 ± 3.42	2.46 ± 0.23
C15	8.75 ± 0.77	40.3 ± 3.88	17.9 ± 1.48
C17	5.16 ± 0.50	30.1 ± 2.76	8.45 ± 0.78
C20	18.4 ± 1.74	56.2 ± 5.48	5.20 ± 0.46

**Table 4:** Hemolytic activities and cytotoxicity of selected compounds.

Compounds	Hemolysis	Cytotoxicity(NIH3T3)
	LC30^a^(mg/mL)	GI50(*μ*M)
C9	>10	199.1 ± 1.60
C10	>10	203.8 ± 3.05
C13	>10	148.5 ± 2.02
C15	>10	154.1 ± 1.75
C17	>10	185.4 ± 3.15
C20	>10	274.4 ± 3.61
DCCP	>10	125.8 ± 3.12

^a^Lytic concentration 30%.
