# Supplementary material for: Design, biological evaluation and 3D QSAR studies of novel dioxin-containing pyrazoline derivatives with thiourea skeleton as selective HER-2 inhibitors
Source: Sci Rep. 2016 Jun 8;6:27571. doi: 10.1038/srep27571 (PMC4897788; doi:10.1038/srep27571)

**Design, biological evaluation and 3D QSAR studies of novel dioxin-containing pyrazoline derivatives with thiourea skeleton as selective HER-2 inhibitors**

Bing Yang^†b^, Yu-Shun Yang^†a, b^, Na Yang^a, b^, Gui-Gen Li*^b^, Hai-Liang Zhu*^a^

^a^ State Key Laboratory of Pharmaceutical Biotechnology, Nanjing University, Nanjing 210093, China

^b^ Institute of Chemistry and BioMedical Sciences, School of Chemistry and Chemical Engineering, Nanjing University, Nanjing 210093, China

^†^ Both authors contributed equally to the work

* Corresponding author. Tel. & Fax: +86-25-8359 2672; E-mail: [zhuhl@nju.edu.cn](mailto:zhuhl@nju.edu.cn); guigenli@nju.edu.cn

**Chemistry section**

**Materials and Measurements**

All commercially available chemicals were used as received without further purification. Melting points were determined on a WRS-1C digital melting point apparatus and were uncorrected. ^1^H NMR, ^13^C NMR and ^19^F NMR spectra were recorded on a Bruker Avance II 400 spectrometer. Shifts were reported in parts per million based on residual solvent peaks (for ^1^H or ^13^C/CDCl_3_). Shifts in ^19^F NMR spectra were reported based on an external hexafluorobenzene reference. NMR data were resolved with MestreNova software. Mass spectra were obtained from an Agilent 6540 UHD Accurate Mass Q-TOF LC/MS.

**General method of synthesis of (*E*)-Chalcones (B)**

To 1-(2,3-dihydrobenzo[*b*][1,4]dioxin-6-yl)ethan-1-one (**A**) (1 mmol) alcohol solution (5 mL) was added substituted benzaldehyde (1 mmol). After dissolution, 50% NaOH (0.5 mL) was added at room temperature. After confirming the completion of the reaction by thin layer chromatography, the sediment was filtered, washed with ethanol and dried to obtain chalcone (**B**).

**General method of synthesis of 3-(2,3-dihydrobenzo[*b*][1,4]dioxin-6-yl)-5-aryl-4,5-dihydro-1*H*-pyrazole-1-carbothioamide (C1-C20)**

Chalcone **B** (0.5 mmol) and thiosemicarbazide (0.5 mmol) in ethanol (5 mL) were refluxed overnight. After the reaction completed, the resultant solid was filtered, washed with ethanol and dried to obtain the corresponding target compound **C**. In some cases, ultrasonic vibration can contribute to precipitation.

**3-(2,3-dihydrobenzo[*b*][1,4]dioxin-6-yl)-5-phenyl-4,5-dihydro-1*H*-pyrazole-1-carbothioamide (C1)**

Yellow solid; mp 166-168 ℃; yield: 83%; ^1^H NMR (400 MHz, CDCl_3_) *δ* 3.14 (d, 1H, *J* = 17.6 Hz), 3.79 (dd, 1H, *J* = 17.6, 11.6 Hz), 4.29 (d, 4H, *J* = 5.6 Hz), 6.01 (d, 2H, *J* = 11.2 Hz), 6.89 (d, 1H, *J* = 8.4 Hz), 7.00-7.35 (m, 8H); ^13^C NMR (100 MHz, CDCl_3_) *δ* 43.3, 63.6, 64.4, 64.7, 116.1, 117.8, 120.8, 124.2, 125.5, 127.7, 129.0, 141.9, 143.8, 146.4, 155.8, 176.5; HRMS (ESI-TOF) m/z: [M+H]^+^ Calcd for C_18_H_18_N_3_O_2_S 340.1114, Found 340.1115; [M+Na]^+^ Calcd for C_18_H_17_N_3_O_2_SNa 362.0934, Found 362.0935.

**3-(2,3-dihydrobenzo[*b*][1,4]dioxin-6-yl)-5-(naphthalen-1-yl)-4,5-dihydro-1*H*-pyrazole-1-carbothioamide (C2)**

Yellow solid; mp 214-216 ℃; yield: 81%; ^1^H NMR (400 MHz, CDCl_3_) *δ* 3.12 (dd, 1H, *J* = 17.2, 3.6 Hz), 3.96 (dd, 1H, *J* = 17.2, 11.2 Hz), 4.24-4.28 (m, 4H), 6.13 (s, 1H), 6.77 (dd, 1H, *J* = 11.6, 7.6 Hz), 6.85 (d, 1H, *J* = 8.4 Hz), 7.16-7.20 (m, 2H), 7.24 (d, 1H, *J* = 2.0 Hz), 7.40 (t, 1H, *J* = 7.6 Hz), 7.50-7.60 (m, 2H), 7.77 (d, 1H, *J* = 8.4 Hz), 7.90 (d, 1H, *J* = 8.0 Hz), 8.00 (d, 1H, *J* = 8.4 Hz); ^13^C NMR (100 MHz, CDCl_3_) *δ* 42.9, 61.1 (d, *J* = 1.0 Hz), 64.3, 64.7, 116.1, 117.8, 120.8, 121.7, 122.9, 124.1, 125.7, 125.9, 126.5, 128.2, 129.4, 129.4, 134.5, 136.3, 143.8, 146.4, 156.5, 176.8; HRMS (ESI-TOF) m/z: [M+H]^+^ Calcd for C_22_H_20_N_3_O_2_S 390.1271, Found 390.1275; [M+Na]^+^ Calcd for C_22_H_19_N_3_O_2_SNa 412.1090, Found 412.1089.

**3-(2,3-dihydrobenzo[*b*][1,4]dioxin-6-yl)-5-(naphthalen-2-yl)-4,5-dihydro-1*H*-pyrazole-1-carbothioamide (C3)**

Yellow solid; mp 204-206 ℃; yield: 87%; ^1^H NMR (400 MHz, CDCl_3_) *δ* 3.20 (dd, 1H, *J* = 17.6, 3.6 Hz), 3.85 (dd, 1H, *J* = 17.6, 11.2 Hz), 4.29 (q, 4H, *J* = 4.8 Hz), 6.03 (s, 1H), 6.18 (dd, 1H, *J* = 11.6, 3.6 Hz), 6.90 (d, 1H, *J* = 8.4 Hz), 7.10-7.32 (m, 4H), 7.41-7.47 (m, 2H), 7.67 (s, 1H), 7.78-7.82 (m, 3H); ^13^C NMR (100 MHz, CDCl_3_) *δ* 43.4, 63.8, 64.4, 64.8, 116.1, 117.9, 120.9, 123.5, 124.2, 124.5, 126.0, 126.4, 127.8, 128.2, 129.1, 133.0, 133.5, 139.3, 143.9, 146.4, 155.8, 176.6; HRMS (ESI-TOF) m/z: [M+H]^+^ Calcd for C_22_H_20_N_3_O_2_S 390.1271, Found 390.1272; [M+Na]^+^ Calcd for C_22_H_19_N_3_O_2_SNa 412.1090, Found 412.1093.

**3-(2,3-dihydrobenzo[*b*][1,4]dioxin-6-yl)-5-(2-fluorophenyl)-4,5-dihydro-1*H*-pyrazole-1-carbothioamide (C4)**

Yellow solid; mp 169-171 ℃; yield: 86%; ^1^H NMR (400 MHz, CDCl_3_) *δ* 3.14 (dd, 1H, *J* = 17.6, 3.6 Hz), 3.81 (dd, 1H, *J* = 17.6, 11.6 Hz), 4.29 (q, 4H, *J* = 5.2 Hz), 6.04 (s, 1H), 6.21 (dd, 1H, *J* = 11.6, 4.0 Hz), 6.89 (d, 1H, *J* = 8.4 Hz), 7.04-7.15 (m, 4H), 7.20-7.26 (m, 3H); ^13^C NMR (100 MHz, CDCl_3_) *δ* 42.2, 58.5 (d, *J* = 3.0 Hz), 64.4, 64.7, 115.8, 116.0, 116.1, 117.8, 120.8, 124.0, 124.4 (d, *J* = 3.0 Hz), 127.3 (d, *J* = 4.0 Hz), 129.4, 129.4, 143.8, 146.4, 156.0, 176.6; ^19^F NMR (376.38 MHz, CDCl_3_) *δ* -117.82; HRMS (ESI-TOF) m/z: [M+H]^+^ Calcd for C_18_H_17_FN_3_O_2_S 358.1020, Found 358.1020; [M+Na]^+^ Calcd for C_18_H_16_FN_3_O_2_SNa 380.0839, Found 380.0838.

**3-(2,3-dihydrobenzo[*b*][1,4]dioxin-6-yl)-5-(3-methoxyphenyl)-4,5-dihydro-1*H*-pyrazole-1-carbothioamide (C5)**

Yellow solid; mp 168-170 ℃; yield: 80%; ^1^H NMR (400 MHz, CDCl_3_) *δ* 3.13 (dd, 1H, *J* = 17.6, 2.8 Hz), 3.72-3.80 (m, 4H), 4.29 (d, 4H, *J* = 4.8 Hz), 5.96-6.00 (m, 2H), 6.74-6.80 (m, 3H), 6.89 (d, 1H, *J* = 8.4 Hz), 6.98-7.26 (m, 4H); ^13^C NMR (100 MHz, CDCl_3_) *δ* 43.3, 55.3, 63.5, 64.4, 64.7, 111.6, 112.8, 116.1, 117.7, 117.8, 120.8, 124.2, 130.1, 143.6, 143.8, 146.4, 155.8, 160.1, 176.6; HRMS (ESI-TOF) m/z: [M+H]^+^ Calcd for C_19_H_20_N_3_O_3_S 370.1220, Found 370.1223; [M+Na]^+^ Calcd for C_19_H_19_N_3_O_3_SNa 392.1039, Found 392.1044.

**5-(3-(benzyloxy)phenyl)-3-(2,3-dihydrobenzo[*b*][1,4]dioxin-6-yl)-4,5-dihydro-1*H*-pyrazole-1-carbothioamide (C6)**

Yellow solid; mp 178-180 ℃; yield: 84%; ^1^H NMR (400 MHz, CDCl_3_) *δ* 3.11 (dd, 1H, *J* = 17.6, 3.6 Hz), 3.75 (dd, 1H, *J* = 17.6, 11.6 Hz), 4.29 (q, 4H, *J* = 4.8 Hz), 5.02 (s, 2H), 5.96-6.00 (m, 2H), 6.82-7.10 (m, 5H), 7.18-7.42 (m, 8H); ^13^C NMR (100 MHz, CDCl_3_) *δ* 43.2, 63.5, 64.4, 64.8, 70.2, 112.4, 113.7, 116.1, 117.8, 118.1, 120.9, 124.2, 127.8, 128.1, 128.7, 130.1, 137.0, 143.6, 143.8, 146.4, 155.8, 159.4, 176.5; HRMS (ESI-TOF) m/z: [M+H]^+^ Calcd for C_25_H_24_N_3_O_3_S 446.1533, Found 446.1535; [M+Na]^+^ Calcd for C_25_H_23_N_3_O_3_SNa 468.1352, Found 468.1356.

**5-(3-chlorophenyl)-3-(2,3-dihydrobenzo[*b*][1,4]dioxin-6-yl)-4,5-dihydro-1*H*-pyrazole-1-carbothioamide (C7)**

Yellow solid; mp 246-248 ℃; yield: 90%; ^1^H NMR (400 MHz, DMSO-*d*_6_) *δ* 3.13 (dd, 1H, *J* = 18.0, 3.2 Hz), 3.82 (dd, 1H, *J* = 18.4, 11.6 Hz), 4.27 (d, 4H, *J* = 3.6 Hz), 5.89 (dd, 1H, *J* = 11.2, 2.8 Hz), 6.91 (d, 1H, *J* = 8.4 Hz), 7.07 (d, 1H, *J* = 7.6 Hz), 7.13 (s, 1H), 7.28-7.37 (m, 3H), 7.44 (d, 1H, *J* = 1.2 Hz), 7.97 (d, 2H, *J* = 43.2 Hz); ^13^C NMR (100 MHz, DMSO-*d*_6_) *δ* 42.2, 62.3, 64.0, 64.4, 115.8, 117.2, 120.9, 124.0, 124.0, 125.2, 126.9, 130.5, 133.0, 143.4, 145.4, 145.7, 154.7, 175.8; HRMS (ESI-TOF) m/z: [M+H]^+^ Calcd for C_18_H_17_ClN_3_O_2_S 374.0725, Found 374.0724; [M+Na]^+^ Calcd for C_18_H_16_ClN_3_O_2_SNa 396.0544, Found 396.0545.

**5-(3-bromophenyl)-3-(2,3-dihydrobenzo[*b*][1,4]dioxin-6-yl)-4,5-dihydro-1*H*-pyrazole-1-carbothioamide (C8)**

Yellow solid; mp 247-249 ℃; yield: 78%; ^1^H NMR (400 MHz, CDCl_3_) *δ* 3.11 (dd, 1H, *J* = 17.6, 3.2 Hz), 3.78 (dd, 1H, *J* = 17.6, 11.6 Hz), 4.29 (d, 4H, *J* = 5.2 Hz), 5.95-6.28 (m, 2H), 6.90 (d, 1H, *J* = 8.4 Hz), 7.00-7.39 (m, 7H); ^13^C NMR (100 MHz, CDCl_3_) *δ* 43.2, 63.0, 64.4, 64.8, 116.2, 117.9, 120.9, 123.1, 123.9, 124.4, 128.6, 130.6, 130.9, 143.9, 144.2, 146.5, 155.6, 176.6; HRMS (ESI-TOF) m/z: [M+H]^+^ Calcd for C_18_H_17_BrN_3_O_2_S 418.0219, Found 418.0223; [M+Na]^+^ Calcd for C_18_H_16_BrN_3_O_2_SNa 440.0039, Found 440.0042.

**3-(2,3-dihydrobenzo[*b*][1,4]dioxin-6-yl)-5-(*p*-tolyl)-4,5-dihydro-1*H*-pyrazole-1-carbothioamide (C9)**

Yellow solid; mp 205-207 ℃; yield: 84%; ^1^H NMR (400 MHz, CDCl_3_) *δ* 2.31 (s, 3H), 3.13 (dd, 1H, *J* = 17.6, 3.2 Hz), 3.76 (dd, 1H, *J* = 17.6, 11.6 Hz), 4.29 (q, 4H, *J* = 4.8 Hz), 5.95-5.99 (m, 2H), 6.89 (d, 1H, *J* = 8.4 Hz), 7.08-7.14 (m, 4H), 7.20-7.26 (m, 3H); ^13^C NMR (100 MHz, CDCl_3_) *δ* 21.3, 43.3, 63.4, 64.4, 64.7, 116.1, 117.8, 120.8, 124.2, 125.5, 129.7, 137.4, 139.0, 143.8, 146.4, 155.8, 176.5; HRMS (ESI-TOF) m/z: [M+H]^+^ Calcd for C_19_H_20_N_3_O_2_S 354.1271, Found 354.1269; [M+Na]^+^ Calcd for C_19_H_19_N_3_O_2_SNa 376.1090, Found 376.1092.

**3-(2,3-dihydrobenzo[*b*][1,4]dioxin-6-yl)-5-(4-methoxyphenyl)-4,5-dihydro-1*H*-pyrazole-1-carbothioamide (C10)**

Yellow solid; mp 186-188 ℃; yield: 85%; ^1^H NMR (400 MHz, CDCl_3_) *δ* 3.13 (dd, 1H, *J* = 17.6, 3.6 Hz), 3.72-3.79 (m, 4H), 4.29 (q, 4H, *J* = 5.2 Hz), 5.94-5.98 (m, 2H), 6.83-7.26 (m, 8H); ^13^C NMR (100 MHz, CDCl_3_) *δ* 43.3, 55.4, 63.1, 64.4, 64.8, 114.3, 116.1, 117.8, 120.8, 124.2, 127.0, 134.2, 143.8, 146.4, 155.9, 159.1, 176.4; HRMS (ESI-TOF) m/z: [M+H]^+^ Calcd for C_19_H_20_N_3_O_3_S 370.1220, Found 370.1222; [M+Na]^+^ Calcd for C_19_H_19_N_3_O_3_SNa 392.1039, Found 392.1039.

**3-(2,3-dihydrobenzo[*b*][1,4]dioxin-6-yl)-5-(4-fluorophenyl)-4,5-dihydro-1*H*-pyrazole-1-carbothioamide (C11)**

Yellow solid; mp 235-237 ℃; yield: 77%; ^1^H NMR (400 MHz, DMSO-*d*_6_) *δ* 3.10 (dd, 1H, *J* = 18.0, 2.8 Hz), 3.81 (dd, 1H, *J* = 18.0, 11.6 Hz), 4.27 (d, 4H, *J* = 4.0 Hz), 5.88 (dd, 1H, *J* = 11.2, 2.4 Hz), 6.91 (d, 1H, *J* = 8.4 Hz), 7.10-7.17 (m, 4H), 7.33 (d, 1H, *J* = 8.4 Hz), 7.44 (s, 1H), 7.93 (d, 2H, *J* = 40.8 Hz); ^13^C NMR (100 MHz, DMSO-*d*_6_) *δ* 42.3, 62.2, 64.0, 64.4, 115.1, 115.3, 115.8, 117.2, 120.8, 124.1, 127.4 (d, *J* = 8.0 Hz), 139.2 (d, *J* = 3.0 Hz), 143.5, 145.7, 154.6, 175.7; ^19^F NMR (376.38 MHz, DMSO-*d*_6_) *δ* -115.98; HRMS (ESI-TOF) m/z: [M+H]^+^ Calcd for C_18_H_17_FN_3_O_2_S 358.1020, Found 358.1022; [M+Na]^+^ Calcd for C_18_H_16_FN_3_O_2_SNa 380.0839, Found 380.0841.

**5-(4-chlorophenyl)-3-(2,3-dihydrobenzo[*b*][1,4]dioxin-6-yl)-4,5-dihydro-1*H*-pyrazole-1-carbothioamide (C12)**

Yellow solid; mp 231-233 ℃; yield: 82%; ^1^H NMR (400 MHz, CDCl_3_) *δ* 3.10 (dd, 1H, *J* = 17.6, 2.8 Hz), 3.78 (dd, 1H, *J* = 17.6, 11.6 Hz), 4.29 (d, 4H, *J* = 5.2 Hz), 5.96-6.05 (m, 2H), 6.89-7.30 (m, 8H); ^13^C NMR (100 MHz, CDCl_3_) *δ* 43.2, 63.0, 64.4, 64.8, 116.1, 117.9, 120.8, 124.0, 127.1, 129.2, 133.5, 140.5, 143.9, 146.5, 155.7, 176.5; HRMS (ESI-TOF) m/z: [M+Na]^+^ Calcd for C_18_H_16_ClN_3_O_2_SNa 396.0544, Found 396.0544.

**5-(4-bromophenyl)-3-(2,3-dihydrobenzo[*b*][1,4]dioxin-6-yl)-4,5-dihydro-1*H*-pyrazole-1-carbothioamide (C13)**

Yellow solid; mp 253-255 ℃; yield: 83%; ^1^H NMR (400 MHz, DMSO-*d*_6_) *δ* 3.09 (dd, 1H, *J* = 18.0, 3.2 Hz), 3.81 (dd, 1H, *J* = 18.0, 11.6 Hz), 4.27 (d, 4H, *J* = 4.0 Hz), 5.85 (dd, 1H, *J* = 11.2, 2.8 Hz), 6.91 (d, 1H, *J* = 8.8 Hz), 7.07 (d, 2H, *J* = 8.4 Hz), 7.32 (dd, 1H, *J* = 8.4, 1.2 Hz), 7.44-7.51 (m, 3H), 7.95 (d, 2H, *J* = 44.4 Hz); ^13^C NMR (100 MHz, DMSO-*d*_6_) *δ* 42.1, 62.3, 64.0, 64.4, 115.8, 117.2, 119.9, 120.8, 124.0, 127.7, 131.4, 142.4, 143.5, 145.7, 154.6, 175.7; HRMS (ESI-TOF) m/z: [M+H]^+^ Calcd for C_18_H_17_BrN_3_O_2_S 418.0219, Found 418.0219; [M+Na]^+^ Calcd for C_18_H_16_BrN_3_O_2_SNa 440.0039, Found 440.0037.

**3-(2,3-dihydrobenzo[*b*][1,4]dioxin-6-yl)-5-(4-iodophenyl)-4,5-dihydro-1*H*-pyrazole-1-carbothioamide (C14)**

Yellow solid; mp 259-261 ℃; yield: 80%; ^1^H NMR (400 MHz, DMSO-*d*_6_) *δ* 3.08 (dd, 1H, *J* = 18.0, 3.2 Hz), 3.80 (dd, 1H, *J* = 18.4, 11.6 Hz), 4.27 (d, 4H, *J* = 4.4 Hz), 5.82 (dd, 1H, *J* = 11.2, 2.4 Hz), 6.92 (t, 3H, *J* = 7.2 Hz), 7.32 (d, 1H, *J* = 8.4 Hz), 7.43 (s, 1H), 7.67 (d, 2H, *J* = 8.0 Hz), 7.94 (d, 2H, *J* = 44.4 Hz); ^13^C NMR (100 MHz, DMSO-*d*_6_) *δ* 42.1, 62.4, 64.0, 64.4, 92.7, 115.8, 117.2, 120.8, 124.0, 127.8, 137.2, 142.9, 143.5, 145.7, 154.6, 175.7; HRMS (ESI-TOF) m/z: [M+H]^+^ Calcd for C_18_H_17_IN_3_O_2_S 466.0081, Found 466.0080; [M+Na]^+^ Calcd for C_18_H_16_IN_3_O_2_SNa 487.9900, Found 487.9901.

**3-(2,3-dihydrobenzo[*b*][1,4]dioxin-6-yl)-5-(4-(methylthio)phenyl)-4,5-dihydro-1*H*-pyrazole-1-carbothioamide (C15)**

Yellow solid; mp 225-227 ℃; yield: 91%; ^1^H NMR (400 MHz, CDCl_3_) *δ* 2.45 (s, 3H), 3.11 (dd, 1H, *J* = 17.6, 3.6 Hz), 3.76 (dd, 1H, *J* = 17.6, 11.2 Hz), 4.31 (q, 4H, *J* = 5.2 Hz), 5.95-5.98 (m, 2H), 6.88-7.26 (m, 8H); ^13^C NMR (100 MHz, CDCl_3_) *δ* 16.0, 43.2, 63.2, 64.4, 64.8, 116.1, 117.9, 120.8, 124.1, 126.2, 127.2, 137.9, 138.9, 143.9, 146.4, 155.8, 176.5; HRMS (ESI-TOF) m/z: [M+H]^+^ Calcd for C_19_H_20_N_3_O_2_S_2_ 386.0991, Found 386.0991; [M+Na]^+^ Calcd for C_19_H_19_N_3_O_2_S_2_Na 408.0811, Found 408.0814.

**5-(4-(benzyloxy)phenyl)-3-(2,3-dihydrobenzo[*b*][1,4]dioxin-6-yl)-4,5-dihydro-1*H*-pyrazole-1-carbothioamide (C16)**

Yellow solid; mp 197-199 ℃; yield: 84%; ^1^H NMR (400 MHz, CDCl_3_) *δ* 3.15 (dd, 1H, *J* = 17.6, 3.2 Hz), 3.77 (dd, 1H, *J* = 17.6, 11.2 Hz), 4.31 (q, 4H, *J* = 4.8 Hz), 5.04 (s, 2H), 5.97-6.00 (m, 2H), 6.91-7.44 (m, 13H); ^13^C NMR (100 MHz, CDCl_3_) *δ* 43.3, 63.1, 64.4, 64.7, 70.2, 115.2, 116.1, 117.8, 120.8, 124.2, 127.0, 127.6, 128.1, 128.7, 134.4, 137.1, 143.8, 146.4, 155.9, 158.4, 176.4; HRMS (ESI-TOF) m/z: [M+H]^+^ Calcd for C_25_H_24_N_3_O_3_S 446.1533, Found 446.1533; [M+Na]^+^ Calcd for C_25_H_23_N_3_O_3_SNa 468.1352, Found 468.1352.

**3-(2,3-dihydrobenzo[*b*][1,4]dioxin-6-yl)-5-(4-(trifluoromethyl)phenyl)-4,5-dihydro-1*H*-pyrazole-1-carbothioamide (C17)**

Yellow solid; mp 252-254 ℃; yield: 75%; ^1^H NMR (400 MHz, CDCl_3_) *δ* 3.11 (dd, 1H, *J* = 18.0, 4.0 Hz), 3.82 (dd, 1H, *J* = 17.6, 11.6 Hz), 4.29 (q, 4H, *J* = 5.2 Hz), 6.04-6.08 (m, 2H), 6.90 (d, 1H, *J* = 8.4 Hz), 7.00-7.26 (m, 3H), 7.33 (d, 2H, *J* = 8.4 Hz), 7.59 (d, 2H, *J* = 8.4 Hz); ^13^C NMR (100 MHz, CDCl_3_) *δ* 43.1, 63.2, 64.4, 64.8, 116.1, 117.9, 120.9, 123.8, 126.0, 126.1 (q, *J* = 3.0 Hz), 126.2, 143.9, 145.8 (d, *J* = 1.0 Hz), 146.6, 155.6, 176.6; ^19^F NMR (376.38 MHz, CDCl_3_) *δ* -62.54; HRMS (ESI-TOF) m/z: [M+H]^+^ Calcd for C_19_H_17_F_3_N_3_O_2_S 408.0988, Found 408.0988; [M+Na]^+^ Calcd for C_19_H_16_F_3_N_3_O_2_SNa 430.0808, Found 430.0806.

**5-(2,6-difluorophenyl)-3-(2,3-dihydrobenzo[*b*][1,4]dioxin-6-yl)-4,5-dihydro-1*H*-pyrazole-1-carbothioamide (C18)**

Yellow solid; mp 208-209 ℃; yield: 81%; ^1^H NMR (400 MHz, CDCl_3_) *δ* 3.24 (dd, 1H, *J* = 18.0, 5.6 Hz), 3.79 (dd, 1H, *J* = 17.6, 12.4 Hz), 4.30 (q, 4H, *J* = 4.8 Hz), 5.96 (s, 1H), 6.20 (dd, 1H, *J* = 12.4, 5.6 Hz), 6.84-7.00 (m, 8H), 7.19-7.26 (m, 3H); ^13^C NMR (100 MHz, CDCl_3_) *δ* 41.2, 59.9 (t, *J* = 2.0 Hz), 64.4, 64.8, 111.6 (d, *J* = 2.0 Hz), 111.8 (d, *J* = 3.0 Hz), 116.1, 117.8, 117.9, 120.7, 124.1, 129.4 (d, *J* = 2.0 Hz), 129.6 (d, *J* = 4.0 Hz), 129.7, 143.8, 146.3, 154.9, 176.1; ^19^F NMR (376.38 MHz, CDCl_3_) *δ* -114.45; HRMS (ESI-TOF) m/z: [M+H]^+^ Calcd for C_18_H_16_F_2_N_3_O_2_S 376.0926, Found 376.0928; [M+Na]^+^ Calcd for C_18_H_15_F_2_N_3_O_2_SNa 398.0745, Found 398.0743.

**3-(2,3-dihydrobenzo[*b*][1,4]dioxin-6-yl)-5-(furan-2-yl)-4,5-dihydro-1*H*-pyrazole-1-carbothioamide (C19)**

Brown solid; mp 174-175 ℃; yield: 72%; ^1^H NMR (400 MHz, CDCl_3_) *δ* 3.40 (dd, 1H, *J* = 17.2, 3.6 Hz), 3.61 (dd, 1H, *J* = 17.2, 11.2 Hz), 4.30 (q, 4H, *J* = 5.2 Hz), 6.00 (s, 1H), 6.11 (dd, 1H, *J* = 11.2, 3.6 Hz), 6.32 (dd, 1H, *J* = 3.2, 1.6 Hz), 6.42 (d, 1H, *J* = 3.2 Hz), 6.91 (d, 1H, *J* = 8.4 Hz), 6.95-7.14 (m, 1H), 7.23-7.29 (m, 3H); ^13^C NMR (100 MHz, CDCl_3_) *δ* 39.3, 57.2, 64.4, 64.8, 108.6, 110.6, 116.2, 117.9, 120.9, 124.1, 142.0, 143.8, 146.4, 152.0, 156.1, 176.4; HRMS (ESI-TOF) m/z: [M+H]^+^ Calcd for C_16_H_16_N_3_O_3_S 330.0907, Found 330.0906; [M+Na]^+^ Calcd for C_16_H_15_N_3_O_3_SNa 352.0726, Found 352.0722.

**3-(2,3-dihydrobenzo[*b*][1,4]dioxin-6-yl)-5-(3,4-dimethoxyphenyl)-4,5-dihydro-1*H*-pyrazole-1-carbothioamide (C20)**

Yellow solid; mp 262-264 ℃; yield: 76%; ^1^H NMR (400 MHz, CDCl_3_) *δ* 3.15 (dd, 1H, *J* = 17.6, 3.6 Hz), 3.71-3.85 (m, 7H), 4.30 (q, 4H, *J* = 5.2 Hz), 5.93-6.24 (m, 2H), 6.74-6.81 (m, 3H), 6.90 (d, 1H, *J* = 8.4 Hz), 7.00-7.26 (m, 3H); ^13^C NMR (100 MHz, CDCl_3_) *δ* 43.3, 56.0 (d, *J* = 4.0 Hz), 63.4, 64.4, 64.8, 109.2, 111.5, 116.1, 117.6, 117.9, 120.8, 124.2, 134.6, 143.9, 146.4, 148.6, 149.4, 156.0, 176.6; HRMS (ESI-TOF) m/z: [M+H]^+^ Calcd for C_20_H_22_N_3_O_4_S 400.1326, Found 400.1327; [M+Na]^+^ Calcd for C_20_H_21_N_3_O_4_SNa 422.1145, Found 422.1150.

Figure S1. Linear regression between the kinase inhibitory activities and the anti-proliferative effect.


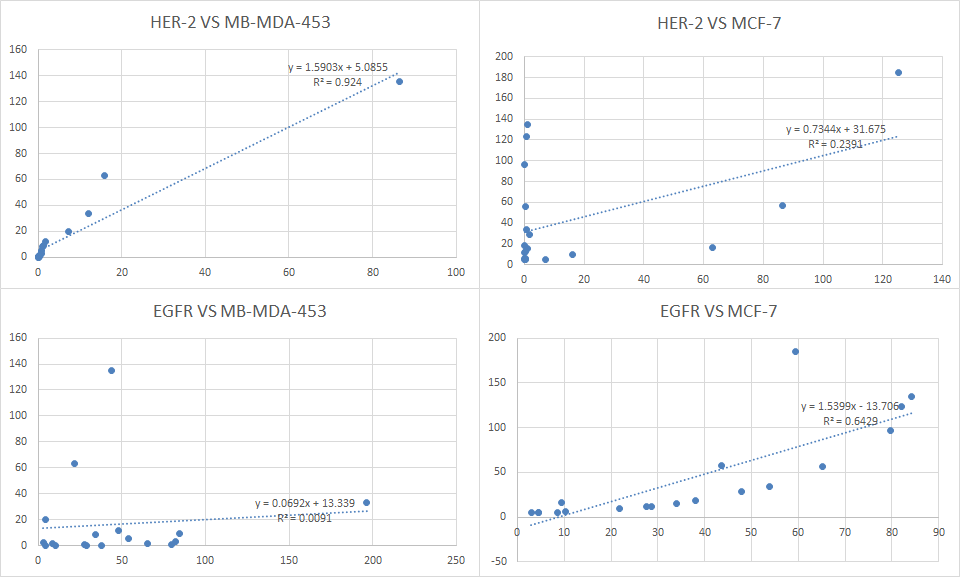


Figure S2. Selectivity of the selected compounds on HER-2, MB-MDA-453, VEGFR-2, HER-3 and FAK.


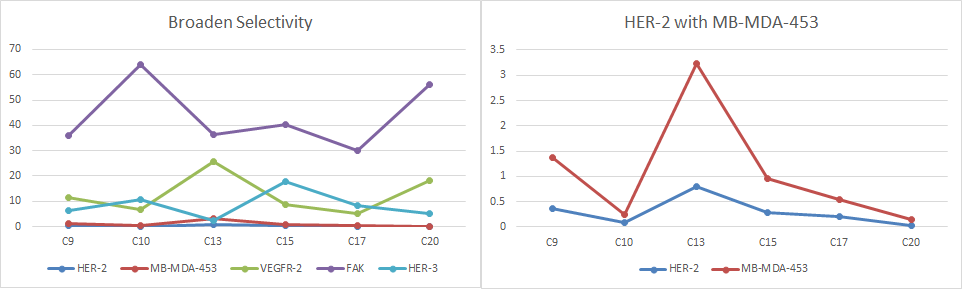


Figure S3. Molecular overlap with benzyloxyphenyl containing compounds (A) and without (B).


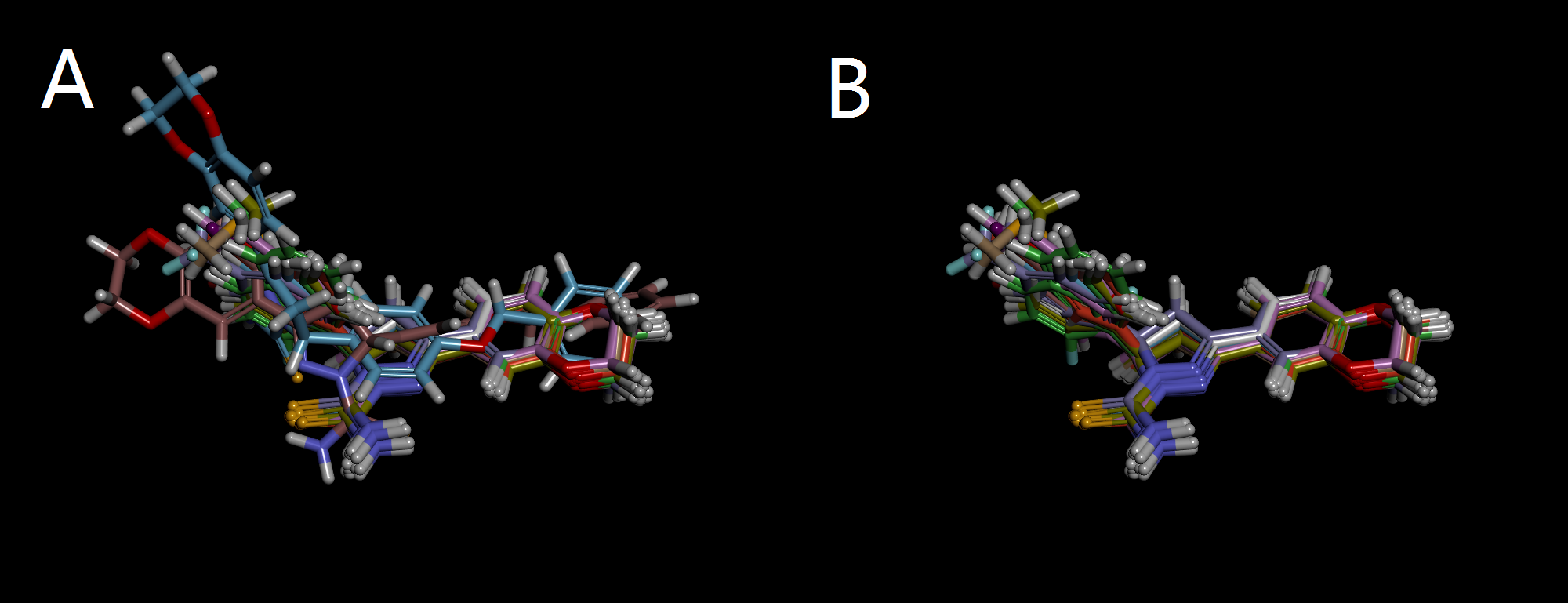

Supplement: Supplementary Information [file srep27571-s1.doc]
